# Evaluating Fitbits for Assessment of Physical Activity and Sleep in Pediatric Pain: Feasibility and Acceptability Pilot Study

**DOI:** 10.2196/59074

**Published:** 2025-07-30

**Authors:** Bridget A Nestor, Andreas M Baumer, Justin Chimoff, Benoit Delecourt, Camila Koike, Nicole Tacugue, Roland Brusseau, Nathalie Roy, Israel A Gaytan-Fuentes, Navil Sethna, Danielle Wallace, Joe Kossowsky

**Affiliations:** 1 Department of Psychology, Endicott College, Beverly, MA, United States; 2 Department of Anesthesiology, Critical Care, and Pain Medicine, Boston Children’s Hospital, 333 Longwood Ave, Boston, MA, 02115, United States, 1 617-355-0965; 3 Harvard Medical School, Boston, MA, United States; 4 Institute for Implementation Science in Healthcare, University of Zürich, Zurich, Switzerland; 5 Department of Cardiac Surgery, Boston Children's Hospital, Boston, MA, United States; 6 OBEDI Obesity Clinic, Mexico City, Mexico; 7 Rollins School of Public Health, Emory University, Atlanta, United States

**Keywords:** Fitbit, pediatric pain, sleep, pediatric, acceptability, feasibility, physical activity, youth, wearable devices

## Abstract

**Background:**

Consumer-grade wearables, such as Fitbits, are a promising, cost-effective methodology for objectively assessing sleep and physical activity in youth with pain.

**Objective:**

This study investigated the acceptability and feasibility of implementing Fitbits for youth with acute and chronic pain in and out of hospital settings while maintaining data security and patient confidentiality.

**Methods:**

We investigated participant experience of Fitbit use over 3 to 4 weeks for a sample of youth with acute pain undergoing either orthopedic or cardiac surgical procedures (N=34, mean age 14.46, SD 3.70 years, 47.06% [n=36] female) and a sample of youth with chronic pain enrolled in an intensive interdisciplinary pain treatment program (N=28, mean age 15.00, SD 2.33 years, 82.14% [n=23] female). We assessed the acceptability of Fitbit use through survey items probing comfort (0=extremely uncomfortable to 10=extremely comfortable), perceived burdensomeness (0=not burdensome at all to 10=extremely burdensome), and open-ended issues or concerns. Feasibility was assessed by tracking the daily compliant wear of the Fitbit device, which was operationalized as more than 600 minutes of daily wear time. We tested for group differences in acceptability and feasibility between orthopedic and cardiac patients within the acute pain sample and between the acute pain and chronic pain samples. We created an automated data pipeline to ensure data security, patient confidentiality, and quality.

**Results:**

Acceptability findings revealed high levels of reported comfort (acute pain: mean 8.56, SD 1.43; chronic pain: mean 8.27, SD 1.69) and low levels of perceived burdensomeness (acute: mean 0.68, SD 1.17; chronic: mean 1.15, SD 1.38) related to Fitbit wearing in both samples. No significant differences in these acceptability outcomes emerged between orthopedic and cardiac patients or between the acute pain and chronic pain groups (*P* values>.10). Transient concerns of mild wrist irritation and sleep discomfort were occasionally reported across both samples (15.79% [n=9] of participants). Feasibility findings indicated high feasibility (acute: median compliance rate of 86.67%; chronic: median compliance rate of 96.65%) for the study duration in both samples. Mann-Whitney U tests indicated significantly higher median compliance rates per participant among orthopedic as compared with cardiac patients (U=146.5, *P*=.04) and significantly higher median compliance rates per participant among the chronic pain group as compared with the acute pain group (U=186, *P*<.001), including significantly higher median compliant days (U=162, *P*<.001) and study days (U=234.5, *P*<.001) per participant.

**Conclusions:**

These findings support the use of Fitbits as an acceptable and feasible method for collecting objective data on sleep and physical activity for youth experiencing pain. Findings also highlight the logistics of implementing consumer-grade wearable devices throughout all stages of the clinical research process.

## Introduction

Pediatric pain affects nearly 40% of youth globally [1-3] and is associated with significant functional impairment across multiple life domains [4]. Youth who develop a chronic pain disorder are also at increased risk for such disorders persisting into adulthood [5], often resulting in costly health care utilization [6] and positioning pediatric pain as a serious public health concern. The gold-standard intervention for pediatric chronic pain assumes an interdisciplinary approach [7], guided and informed by the biopsychosocial pain model [8], where treatment aims to address multifactorial influences on pain [9].

Physical activity and sleep are 2 biopsychosocial factors critical to the experience of pediatric pain and are identified as core outcome domains for its treatment and study [6]. Prior research has identified bidirectional relations between decreased physical activity and increased pain [10] and between decreased sleep duration and quality and increased pain [11,12]. A reliable and valid assessment of physical activity and sleep is necessary for intervention efforts in pediatric pain. Such assessment must also accurately and precisely monitor and measure changes in these constructs over time (ie, improvements and deteriorations in physical activity and sleep).

New multisensory, consumer-facing technology has emerged as a promising means to continuously assess physical activity and sleep in research [13]. Of these consumer-grade devices, Fitbits have garnered the most research [14] and are significantly more affordable, widely available, and easy to use, highlighting their potential for large-scale studies and long-term monitoring. Fitbits capture physical activity and sleep metrics using accelerometer data collected in 1-minute and 30-second epochs, respectively [15,16]. Technologically, newer Fitbits differ from actigraphy devices in that they leverage multisensory capabilities, differentiating them from actigraphy devices, which primarily use accelerometer data. For example, Fitbits incorporate an optical heart rate sensor in addition to accelerometry, allowing for more refined sleep staging capabilities by distinguishing between light, deep, and REM sleep phases. With this new multisensory technology, newer versions of Fitbit devices can also accurately capture sleep stages and heart rate and demonstrate a specificity of 61%‐69% [17], which is significantly higher than that of actigraphy [16]. In contrast, traditional actigraphy devices estimate sleep solely based on movement patterns, often resulting in lower specificity.

Fitbits have high user acceptability over long periods of time [18], and have demonstrated high levels of accuracy and sensitivity for correctly identifying sleep episodes compared with polysomnography [19], a laboratory-based sleep methodology that leverages simultaneous recordings of multiple physiological indicators, including brain activity, eye movements, heart rate, and breathing patterns. Findings show that Fitbits provide measures of total sleep time that are statistically indistinguishable from polysomnography - and actigraphy-measured total sleep time [20], including in pediatric samples [21-23]. Fitbits also boast cost-effective prices, and as wrist-worn devices, are noninvasive methodologies. Accordingly, these methodological strengths position Fitbits as a scalable method. Indeed, large-scale, longitudinal, multi-site studies funded by the National Institutes of Health, such as the Adolescent Brain Cognitive Development study, have incorporated Fitbits into their data collection for youth [21]. Other research has begun to examine the use of Fitbits to assess physical activity or sleep in youth [24,25], both in healthy samples [26], as well as those with chronic health conditions [27,28] and physical and developmental disabilities [29,30]. Investigations in pediatric pain, however, have been minimal. With an exception, a recently published pilot study examined the feasibility of using a much older iteration of Fitbit for assessing physical activity and sleep in adolescents at an intensive interdisciplinary pain rehabilitation program [31]. However, the data from this study were somewhat incomplete due to challenges with compliance, and the device used did not incorporate multisensory technology.

Further investigation is needed to use Fitbits in pediatric pain populations for several reasons. First, from a clinical perspective, reliable and valid physical activity and sleep assessment are integral to treatment progress for acute and chronic pediatric pain populations [32]. Second, from a research perspective, a more detailed description of the practical issues and challenges relevant to the research implementation of Fitbit is critical for developing best practices for using consumer-grade methodologies, particularly those with proprietary, “black-box” algorithms [14]. Third, more research using Fitbits is needed to augment and contrast other large-scale adolescent studies already underway that have adopted Fitbit data collection (eg, Adolescent Brain Cognitive Development ).

Therefore, the current article describes our implementation of Fitbits for assessing physical activity and sleep in samples of youth with acute and chronic pain – those undergoing orthopedic or cardiac surgery (acute pain) and those in an interdisciplinary pain rehabilitation program (chronic pain). We chose to investigate 2 samples of surgical patients with acute pain to determine the extent to which Fitbits could be acceptably and feasibly implemented in varied surgical populations. Similarly, we chose to investigate an acute and a chronic sample to assess the extent to which Fitbit’s acceptability and feasibility may vary by pain acuity and patient care environment. We hypothesized that Fitbits would be acceptable and feasible devices for the assessment of physical activity and sleep in youth with acute and chronic pain. We further hypothesized that greater acceptability and feasibility would be observed in youth with chronic pain, as opposed to acute pain, due to the inherent challenges associated with inpatient and postoperative care. These challenges may range from the clinical workflow of the surgical process to difficulty wearing a Fitbit with surgical wounds or drains to potential reliance on medical personnel for refitting the Fitbit after surgery.

Methodologically, we explain our quantitative methods for determining the acceptability and feasibility of Fitbit assessment in this population, and we also explain narratively our process of Fitbit research implementation, including data collection and data management. We then present acceptability and feasibility data collected from our samples. Finally, we discuss our viewpoints on the research implementation of Fitbits in pediatric populations with pain and provide our recommendations for best practices of this assessment method for future pediatric research.

## Methods

### Ethical Considerations

Institutional Review Board (IRB) of Boston Children’s Hospital approved this study of the 3 patient groups (cardiac surgery: IRB-P0003441, April 12, 2021; orthopedic surgery: IRB-P00038993, July 27, 2021; chronic pain: IRB-P00036475, October 07, 2021). Informed consent was obtained. Privacy and confidentiality of research participants’ data and identity were maintained. Compensation varied by study protocol: acute pain participants received US $25, and chronic pain participants received US $90 as part of a comprehensive parent study.

### Participants

Two samples of participants seeking pain treatment services for acute or chronic pain were recruited from a tertiary pediatric hospital in the Northeast United States. The first sample consisted of youth with acute pain who were recruited before undergoing either cardiac or periacetabular osteotomy orthopedic surgery (acute pain group). The second sample consisted of youth with disabling chronic musculoskeletal pain who were enrolled in an outpatient intensive interdisciplinary pain treatment (IIPT) program (chronic pain group), which operates on a day-hospital model of rehabilitation.

Participants were included in the study if they were (1) between 8 and 18 years of age, (2) willing to wear a Fitbit watch, and (3) requiring pain treatment services immediately following cardiac surgery, periacetabular osteotomy orthopedic surgery, or disabling chronic pain. Participants were excluded for intellectual disability. All participants understood and responded to questions in English. Data collection occurred from February 2022 to February 2024.

Eligible patients were identified by research staff through weekly screening of clinic schedules. A research staff contacted the caregiver or legal guardians of identified patients via mail, email, or secure patient portal message with information about the study. Several days after the information was sent, patients were contacted via phone or in the clinic to explain the study requirements and answer any questions. Patients who expressed interest were then consented to the study.

The total acute pain group consisted of 34 participants (mean age 14.46, SD 3.70 years, 47.06% [n=16] female) who provided acceptability or feasibility data. Of these 34 patients, 24 were cardiac patients (mean age 12.92, SD 2.24 years, 33.33% [n=8] female), and 10 were orthopedic patients (mean age 18.30, SD 3.92 years, 80.00% [n=8] female). Some participants in the acute pain group withdrew from the study due to changes in the scheduling of surgery and location of family prohibiting sending/returning the device. The total chronic pain group consisted of 28 participants (mean age 15.00, SD 2.33 years, 82.14% [n=23] female) who provided acceptability and feasibility data. Participants in the chronic pain group who withdrew from study participation cited illness (ie, contracting COVID-19), lack of interest in the study, schedule changes, and withdrawal from IIPT as reasons for discontinuing their participation. Notably, one participant withdrew from participation due to getting a rash from the device. Missing Fitbit data was due only to participants not wearing the device.

### Participant Onboarding Procedures

#### Acute Pain Group

For patients with adequate time before their planned scheduled preoperative visit or date of surgery (DOS), a research staff member obtained written informed consent from those who provided remote consenting (eConsenting) on REDCap (Research Electronic Data Capture) [33] via phone call or Zoom. Patient assent and parental consent were obtained via electronic signature on REDCap. When patients were scheduled close to a preoperative visit or DOS, a research staff member obtained written assent from patients and consent from their parent/guardian on the preoperative visit day, typically the day before or a few days before the DOS.

Surgical patients received a Fitbit device and account information at the end of their consent meeting on their preoperative visit day. Those who consented via eConsenting received a mailed Fitbit device and account information. After receiving their device in person or via mail, a research staff member contacted the families via Zoom to explain the Fitbit setup and answer any questions. Participants were instructed to wear their Fitbit continuously throughout the study. Specifically, they were asked to wear their Fitbit into the hospital on their DOS; a research staff member removed the device before the surgery and replaced it after. Participants were allowed to keep their Fitbit on during bathing and showering, if desired, and they could take small breaks as needed. Participants were also instructed to sync their devices every 2 to 3 days and charge them every 7 days.

During the study, participants indicated their understanding of device setup procedures, and they were given a copy of the standard operating procedures (SOPs) when they received their Fitbit device (either in person or via mail). Multimedia Appendix 1 contains the SOP of Fitbit configuration process. Participants set up their devices independently with support from the SOPs. Contact information for the research staff was provided if families requested additional support, either via phone call or Zoom meeting.

#### Chronic Pain Group

Patients who expressed interest during the recruitment phone call were informed that they would be consented on the admission day to the outpatient IIPT program. On the first admission day, a research staff member obtained written informed assent from patients and consent from their parents/guardians. On the second day, these patients were provided with Fitbit devices and account information. This onboarding was not completed during the patients’ first day of admission due to the busy admission process by different disciplines at the IIPT. A research staff member explained the Fitbit setup, answered any questions, and provided the same standard instructions to sync their device every 2 to 3 days and charge it every 7 days. Multimedia Appendix 1 contains the SOP of Fitbit configuration process.

#### Fitbit Account Creation and User Agreements

The Fitbit setup process included creating a participant account. Before onboarding, the study team created a unique deidentified email account for each new participant. A unique email account was provided to each newly enrolled participant when registering/joining Fitbit during the study setup.

### Instruments and Measures

#### Fitbit Charge 3

The Fitbit Charge 3 (Fitbit Inc; 2018)was implemented for the cardiac surgical participants in the acute pain group. The Fitbit Charge 3 is a wrist-worn consumer-grade device that uses multisensory technology to provide relevant physical activity and sleep metrics. For physical activity, the Fitbit Charge 3 records steps taken and mileage with its 3D accelerometer, allowing motion and acceleration tracking in all directions. For sleep assessment, it leverages accelerometer technology and a PPG sensor to calculate heart rate, monitor sleep patterns, and identify sleep stages (eg, rapid eye movement and nonrapid eye movement).

#### Fitbit Inspire 2

The Fitbit Inspire 2 (Fitbit Inc; 2020) was implemented for orthopedic surgical and chronic pain group participants. Like the Fitbit Charge 3, the Inspire 2 also assesses heart rate and provides the same activity metrics of interest (ie, step count) and sleep-tracking capabilities. The newer Fitbit Inspire 2 model offers a longer battery life and a slightly thinner and lighter build than the Fitbit Charge 3. Both devices use the same sleep assessment algorithm.

#### Acceptability

Acceptability was assessed through self-report scales and open-ended questions developed by the research team. Specifically, on a 10-point scale, participants rated their comfort (“How comfortable was it to wear the watch?” 0=extremely uncomfortable to 10=extremely comfortable) and burden (“How much of a burden/chore was it to wear the watch?” 0=not burdensome at all/not a chore at all to 10=extremely burdensome/extreme chore). Participants in the chronic pain group were additionally asked on the extent to which wearing the Fitbit disturbed their sleep on a 10-point scale (“How much did wearing the Fitbit disturb your sleep?” 0=no disturbance at all to 10=extremely disturbed). All participants were also asked open-ended questions about potential adverse reactions from wearing the Fitbit (“Did you have any adverse reactions or experience any other distressing issues while wearing the Fitbit?”) and about how much they enjoyed participating in the study.

#### Feasibility

This study determined feasibility by participant compliance with wearing the Fitbit, as indicated by device use. Consistent with other thresholds defined for wrist-worn consumer wearable assessment in biobehavioral research, a 600-minute cut-off was implemented to determine daily compliant use for activity tracking [34]. Participants were considered compliant if they wore their device for 600 minutes or more during the day, excluding sleep periods, and for 180 continuous minutes or more during the night while asleep, without off-wrist indicators. Compliance assessment was facilitated by minute-level heart rate data from Fitbit, based on the assumption that recorded heart rate data signified the device was worn. If the heart rate sensor was deactivated or malfunctioned, the Fitbit would still register activity data. Thus, minutes without both heart rate and activity data were assumed to be times the device was not worn, signifying noncompliance.

Compliance ratings for the total number of compliant days out of the total number of study days were generated for the acute pain group and the chronic pain group as described above. Due to variability in the time of day that participants began and stopped wearing their devices on the first and last day of the study, actigraphy data were trimmed to exclude the first and last partial day of measurement. Similarly, sleep data were trimmed to exclude the first night of measurement. Compliance rates throughout the study were computed for each participant and each sample.

### Study Timeline

#### Acute Pain Group

After receiving their Fitbit device at the preoperative appointment, participants in the acute pain group were instructed to continuously wear their Fitbit before their surgery, immediately after their surgery, while inpatient, and for 2 weeks after discharge. Devices were not worn during the surgical procedures. Participants answered acceptability and feasibility survey questions as part of a semistructured interview with a research staff member at the end of their study participation.

#### Chronic Pain Group

After receiving their Fitbit device on day 2 of IIPT, participants in the chronic pain group were instructed to continuously wear their Fitbit devices for the duration of their admission to the IIPT program. Treatment duration in this program is typically 4 weeks (8 h/day, 5 d/week), and participants were also instructed to wear the devices on the weekends. Participants completed the acceptability and feasibility survey items at the end of their study participation at their discharge.

### Data Collection and Processing

**Figure 1.**
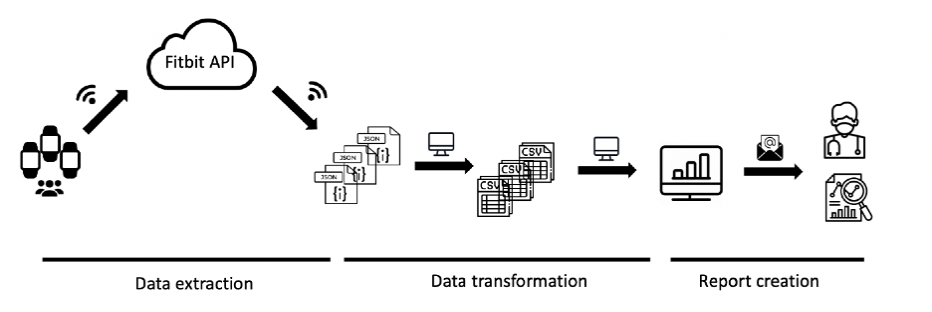
Visual depiction of the automated Fitbit data pipeline. First, raw Fitbit data from each participant is extracted via API calls and stored locally in an unstructured JSON format. Second, these raw JSON files are processed and transformed into structured tabular CSV files for analysis. Third, the pipeline generates automated reports with data visualizations, enabling both researchers and participants to monitor compliance and review summarized activity and sleep trends. API: application programming interface; CSV: Comma-Separated Values; JSON: JavaScript Object Notation.

Because no prior automated process was available for data collection using Fitbits for research purposes in our hospital, we created an automated pipeline using Python (version 3.11; Python Software Foundation) to securely collect and manage participant device data (Figure 1). This pipeline enacted 3 steps: data extraction, transformation, and report creation.

Our pipeline was initiated by connecting to the participants’ accounts using the provided anonymous emails and passwords. Data was then requested via the Fitbit web application programming interface, storing the resulting JSON data files on our secure hospital servers. These JSON files were then reformatted into structured, tabular CSV files, encapsulating relevant sleep, heart rate, and activity metrics at either 30-second or 1-minute intervals, from which daily summary statistics were derived. Subsequently, the pipeline generated reports featuring data visualizations to monitor compliance and provide participants with an intuitive summary of their health data (detailed in the Results section). This process was automated to iterate for all active participant Fitbit accounts. To ensure ongoing participant compliance, a research staff member performed daily reviews of the generated reports. This allowed for the identification and subsequent contact of participants who either failed to sync their devices for 3 consecutive days or exhibited 2 consecutive days of compliance issues, to provide reminders and troubleshooting support.

### Statistical Analysis

Analyses conducted in this study were largely descriptive. For acceptability outcomes, participants’ self-reported device comfort, perceived burdensomeness, and sleep disturbance were summarized for means and standard deviations. Adverse reactions and responses to open-ended inquiries were reported descriptively. For feasibility outcomes, compliance rates were summarized using medians and ranges. Tests of differences using independent samples *t* tests were conducted to investigate mean differences in acceptability between acute pain and chronic pain groups, as well as between orthopedic and cardiac samples. Similarly, Mann-Whitney U tests were conducted to examine median differences in compliance between acute pain and chronic pain groups and between orthopedic and cardiac samples.

## Results

### Acceptability

#### Overview

**Figure 2.**
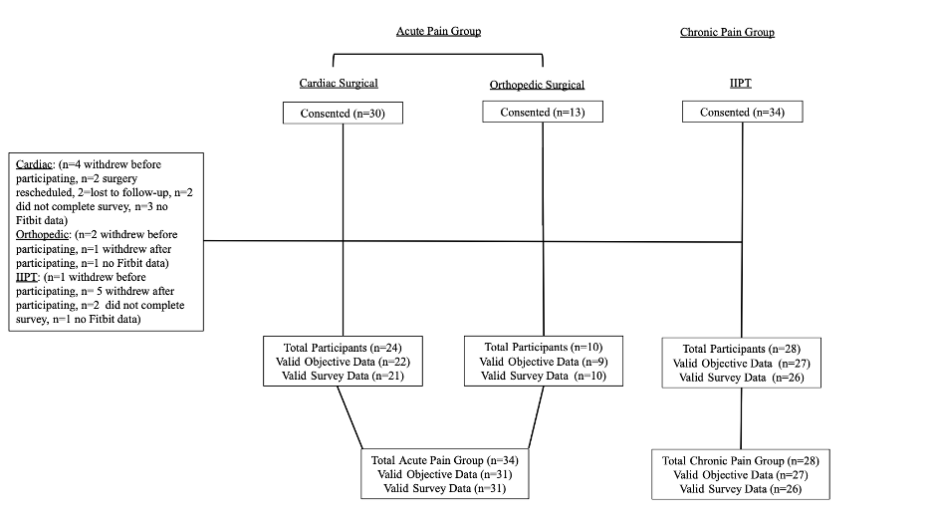
CONSORT diagram of participants included in the study. IIPT: intensive interdisciplinary pain treatment.

A total of 31 participants in the acute pain group and 26 participants in the chronic pain group completed items related to acceptability. Findings indicated high levels of comfort and low levels of burdensomeness reported by participants in both samples and very low levels of sleep disturbance in the chronic pain group (Table 1). Figure 2 depicts the CONSORT diagram.

Results of independent samples *t* tests (Table 1) indicated no significant differences in participant comfort or perceived burdensomeness between orthopedic and cardiac samples or between the acute pain and chronic pain groups (*P* values>.10).

**Table 1.** Participant reported comfort with and the burdensomeness of wearing Fitbit devices.

	Acute pain					Chronic pain		
	Orthopedic (N=10), mean (SD)	Cardiac (N=21), mean (SD)	Test of mean differences^a^		Total (N=31), mean (SD)	IIPT^b^ (N=26), mean (SD)	Test of mean differences^c^	
*t* test (*df*=29)	*P* value	*t* test (*df*=55)	*P* value
Comfort^d^	8.80 (1.14)	8.48 (1.60)	−0.57	.58	8.56 (1.43)	8.27 (1.69)	−0.71	.49
Burden^e^ /Chore	0.30 (0.48)	0.86 (1.35)	1.27	.22	0.68 (1.17)	1.15 (1.38)	1.39	.17
Sleep disturbance^f^	NA^g^	NA	NA	NA	NA	0.69 (1.12)	NA	NA

a Independent samples *t* test comparing orthopedic and cardiac samples.

b IIPT: intensive interdisciplinary pain treatment.

c Independent samples *t* test comparing Acute Pain and Chronic Pain [35].

d Comfort was assessed on a 0‐10 scale (0=extremely uncomfortable to 10=extremely comfortable).

e Burdensomeness was assessed on a 0‐10 scale (0=not burdensome at all/not a chore at all to 10=extremely burdensome/extreme chore).

f The degree to which the Fitbit disturbed their sleep was assessed on a 0‐10 scale (0=no disturbance at all to 10=extremely disturbed).

g NA: not assessed.

#### Adverse Reactions

In the acute pain group, 90.3% (28/31) reported no adverse reactions or distressing issues related to their Fitbit wear. In the chronic pain group, 76.9% (20/26) reported no adverse reactions or distressing issues while wearing their Fitbit. For participants in both groups who did report adverse reactions or other distressing issues, responses are presented in Textbox 1.

Textbox 1. **Participants reported adverse reactions and other distressing issues while using Fitbits.**

Acute pain (N=3)

- “Sometimes put on too tight; user error” [Cardiac sample]
- “Hot during sleep, may be due to tightness on wrist” [Cardiac sample]
- “Once after shower it was red” [Orthopedic sample]

Chronic pain (N=6)

- “Got a rash after taking it off”
- “Brightness of watch when falling asleep”
- “Yes, a mild rash on left side. Removed watch and put it on right side”
- “My wrist got slightly irritated”
- “Sometimes it left a mark on my skin but it was fine”
- “Slight red irritation, dry skin, not itchy/painful”

#### Open-Ended Responses

Responding to open-ended inquiries, some participants reported that wearing the Fitbit device was a positive experience. For example, participants in the acute pain group reported they “liked the Fitbit,” that “the Fitbit was cool,” that “the Fitbit was very fun,” and that it was “fun to have [the] watch.” Similarly, other participants in the chronic pain group reported that it was “kind of fun, nice seeing how well I slept and heart rate,” that they “liked wearing fitbit, helpful to use it,” that they “liked to use the Fitbit and the phone,” and that they “liked wearing [the] fitbit and seeing data.*”*

Other participants provided responses that indicated issues with wearing the Fitbit device. For example, participants in the acute pain group reported that “sleeping in the Fitbit and wearing the Fitbit started to be uncomfortable for a little bit,” that they “sometimes forget to put on a watch, like a secondary Apple watch,” and that “Fitbit hurt during sleep because I move around a lot and put the watch tight.” Participants in the chronic pain group reported that it was “annoying sometimes that there weren’t any breaks from wearing it” and that they “kept losing [their] watch.”

### Feasibility

In the acute pain group, compliance data were obtained from 31 participants (22 cardiac surgical participants and 9 orthopedic surgical participants). All participants in the acute pain group had 604 total days of study duration. Of those, 485 days were classified as compliant per the 600-minute threshold. The median compliance rate for the acute pain group was 86.67%.

In the chronic pain group, compliance data were obtained from 27 participants. There were 624 total days of study duration across all participants in the chronic pain group. Of those, 583 days were classified as compliant per the 600-minute threshold. The median compliance rate was 95.65% for the chronic pain group. Table 2 contains additional details about compliance rates for both samples.

Results of Mann-Whitney U tests (Table 2) indicated significantly higher median compliance rates per participant among orthopedic as compared with cardiac samples (U=146.5, *P*=.04). Results also indicated significantly higher median compliance rates per participant among the chronic pain group as compared with the Acute Group (U=186, *P*<.001), including significantly higher median compliant days (U=162, *P*<.001) and study days (U=234.5, *P*<.001) per participant.

In the acute pain group, sleep compliance data were obtained from 31 participants (22 cardiac surgical participants and 9 orthopedic surgical participants). All participants in the acute pain group had 524 total days of study duration. Of those, 478 days were classified as compliant per the 180-minute threshold. The median compliance rate for the acute pain group was 90.91%.

In the chronic pain group, compliance data were obtained from 27 participants. There were 631 total days of study duration across all participants in the chronic pain group. Of those, 596 days were classified as compliant per the 180-minute threshold. The median compliance rate was 95.65% for the chronic pain group. Table 3 contains additional details about compliance rates for both samples.

Results of Mann-Whitney U tests (Table 3) did not indicate significant differences in median compliance rates per participant among orthopedic as compared with cardiac samples (U=338, *P*=.20).

As noted, compliance monitoring was enacted through daily checks of generated reports to identify participants who had not synced their devices in 3 days or demonstrated compliance rates beneath the threshold of 600 minutes of daily wear time. These reports also provided visualizations of both sleep and physical activity (Multimedia Appendix 1).

**Table 2.** Day compliance data during the study period. For 3 participants in the acute pain group, device-wearing did not begin until postdischarge due to medical complications during hospitalization.

	Acute pain					Chronic pain		
	Orthopedic (N=9)	Cardiac (N=22)	Test of median differences^a^		Total (N=31)	IITP^b^ (N=27)	Test of median differences^c^	
U test	*P* value	U test	*P* value
Number of compliant days per participant, median (range)	18 (11-29)	15.5 (6-29)	128.5	.20	16 (6-29)	22 (9-27)	162.5	<.001
Number of study days per participant, median (range)	19 (14-29)	21 (11-30)	96	.91	20 (11-30)	24 (10-18)	234.5	<.001
Total number of compliant days	160	325	—^d^	—	485	583	—	—
Total number of study days	175	429	—	—	604	624	—	—
Compliance rate per participant (%), median (range)	90 (78.57‐100)	80 (36.36‐96.67)	146.5	.04	86.67 (36.36‐100)	95.65 (60‐100)	186	<.001

a Mann-Whitney U test comparing orthopedic and cardiac samples.

b IIPT: intensive interdisciplinary pain treatment.

c Mann-Whitney U test comparing Acute Pain vs Chronic Pain [36].

d Not applicable.

**Table 3.** Sleep compliance data during the study period. For 3 participants in the acute pain group, device-wearing did not begin until postdischarge due to medical complications during hospitalization.

	Acute pain					Chronic pain		
	Orthopedic (N=9)	Cardiac (N=22)	Test of median differences^a^		Total (N=31)	IITP^b^ (N=27)	Test of median differences^c^	
U test	*P* value	U test	*P* value
Number of compliant nights per participant, median (range)	17 (8-30)	12.5 (4-29)	84	.53	13 (4-30)	23 (5-29)	200	<.001
Number of study nights per participant, median (range)	20 (9-30)	14.5 (6-31)	87.5	.63	15 (6-31)	23 (8-29)	191.5	<.001
Total number of compliant nights	156	322	—^d^	—	478	596	—	—
Total number of study nights	166	358	—	—	524	631	—	—
Compliance rate per participant (%), median (range)	90 (80.96‐100)	92.23 (55.56‐100)	85	.55	90.91 (55.5‐100)	95.65 (62.50‐100)	338	.20

a Mann-Whitney U test comparing orthopedic and cardiac samples.

b IIPT: intensive interdisciplinary pain treatment.

c Mann-Whitney U test comparing Acute Pain vs Chronic Pain [36].

d Not applicable.

## Discussion

This study investigated the implementation of Fitbits for assessing physical activity and sleep in youth with acute and chronic pain. Consistent with our hypotheses, our findings position Fitbits as acceptable and feasible methods of assessment in these populations. To our knowledge, this is the first study investigating the acceptability and feasibility of using Fitbits in youth with pain after surgery and in outpatient rehabilitation settings. Below, we discuss our findings, highlight the logistical challenges of this work, and suggest avenues for future research.

In both acute and chronic pain groups of this study, Fitbits garnered similar and high levels of acceptability from youth. Adolescents in both samples and across both Fitbit devices reported a high degree of comfort and a very low degree of burdensomeness with wearing their devices, echoing prior research that has also found high levels of acceptability for wearable trackers in other pediatric samples [25,37-39]. Open-ended responses also highlighted adolescents’ positive experiences with Fitbit-wearing. For example, youth reported that they enjoyed wearing their Fitbit, that it was fun to have their device, and that they enjoyed raising awareness of their sleep pattern and physical activity behaviors. This highlights the potential for using this method for intervention to help youth self-monitor and improve their own sleep and activity levels, which in turn could improve their ability to manage their pain.

Adolescents’ positive perspectives on using Fitbits could have been related to the youth’s general enthusiasm for technology [40]. For pediatric samples with pain, our study’s positive responses to Fitbits may have reinforced adaptive health-focused behaviors to engage in clinical treatment and monitor functional progress, such as sleep hygiene, activity pacing, behavioral activation, etc. On the other hand, the few negative responses may indicate low tolerance or concerns about discomfort at night, minor skin irritations, and the device’s light emission, which have been reported in other pediatric populations [41]. Future studies using Fitbits in youth may benefit from addressing these concerns and specifically emphasizing appropriate tightness of the device, adequate cleanliness of the band, and brief removal of the device after periods of extended wear. Additionally, it would be important to guide patients on how to adjust relevant Fitbit settings, for example, dimming the display brightness to not interfere with sleep and circadian rhythms, and ensuring the heart rate sensor is activated.

In addition to acceptability, our results also highlight Fitbit’s feasibility as an assessment method for youth with pain. Despite some variability, particularly in our cardiac patients, these results showed high median compliance rates of over 85% across youth with acute and chronic pain. These findings indicate higher compliance rates than were observed in a recently published feasibility study using a much older iteration of Fitbit for assessing physical activity and sleep in adolescents at a similar intensive interdisciplinary pain rehabilitation program [31]. However, it is also worth noting that compliance rates were significantly higher for participants enrolled in IIPT than for the surgical samples, which was consistent with our hypotheses. In this study, the lower compliance rate for surgical patients, as compared with chronic patients, might be related to the clinical workflow of the surgical process, postoperative inpatient setting, patients’ distress with pain, anxiety, caring for the surgical wound/drains, and poor sleep, particularly after cardiac surgery, and reliance on medical personnel for refitting the Fitbit after surgery. Indeed, our findings also revealed significantly lower compliance rates for cardiac patients as compared with orthopedic patients, likely due to the extra challenges and stressors inherent to cardiac versus orthopedic surgery. In general, compliance rates should be interpreted cautiously, particularly following surgical procedures, as medical necessities may override device compliance during this critical period, such as intravenous therapy, invasive monitoring lines, extended ICU stays, etc. It should also be noted, in this study, that orthopedic (and chronic pain) patients wore the Fitbit Inspire 2, whereas cardiac patients wore the Fitbit Charge. Although both devices use similar assessment technology, the Inspire 2 has a thinner and sleeker design, which may be relevant to these differential compliance rates.

Several practical challenges and limitations emerged that are relevant to the feasibility of using Fitbits for research purposes. First, the Fitbit device requires participants to charge it approximately every 7 days. In this study, the period of device charging was a particularly risky window for low compliance, suggesting that a reminder strategy may help youth replace their device after charging. Fortunately, compared with other consumer-grade wearable devices, Fitbits have a much longer battery life and thus require less frequent charging (eg, compared with Apple Watch, which has a shorter 18 h battery life). Failure to sync the device promptly posed a further challenge for data collection. Because of limited storage space on Fitbit devices, it can hold high-resolution (ie, minute or second) data for about 7 days, with the exact duration varying across different models. If the device is not synced in this timeframe, it will discard the oldest high-detail information and keep only the daily summary values. Participants were instructed to sync their devices at least every 3 days to ensure we had access to the highest possible data resolution. Our research team reached out to the participants with low compliance data and low battery status, as indicated by daily checks of the data pipeline. Second, we found that specific Fitbit settings must be activated for proper data collection. For example, Fitbits can be worn on the wrist or a clip. However, when the device is switched to “clip mode,” heart rate and sleep are not recorded due to the deactivation of the optical PPG sensor (skin sensor). For one participant in the Chronic Pain Sample, the Fitbit was inadvertently switched into “clip mode,” resulting in the loss of 2 weeks’ worth of data. This highlights the importance of providing clear instructions to participants and periodic reminders on the proper use of the device for data fidelity. To address these challenges, we suggest that future work develop comprehensive SOPs to avoid the recurrent compromising of data. Third, we acknowledge that the Fitbit Charge 3 was released in 2018, making it not the most current device. However, in clinical practice, many institutions and families still use these devices due to cost and accessibility. Additionally, prior to launching the acute pain study, we conducted a pilot trial to assess the initial acceptability and feasibility of the Fitbit Charge 3 within this population. Based on the positive findings from that pilot, as well as the prior institutional purchase of these devices, we chose to maintain consistency by continuing to use the Charge 3. This approach minimized variability in data collection while also leveraging existing resources to enhance study feasibility. Finally, given the nature of the IIPT as a day program with close contact with study personnel over the course of 4 weeks, we, unfortunately, could not control for differences in the amount of contact with study staff and its potential influence on our findings.

Ensuring patient confidentiality and the highest possible level of transparency during patient consent was also central to the feasible implementation of Fitbits in this study. We took several measures to ensure this throughout the study duration in close communication with our IRB. First, we created deidentified Fitbit accounts and linked them to anonymous email addresses only accessible by the research team. The account information was stored in private, protected folders on central computers at Boston Children’s Hospital, as the Privacy Rule of the Health Insurance Portability and Accountability Act (HIPAA) recommended. Second, each new participant was required to agree to the end user license agreement of Fitbit during participant onboarding. In addition, we used devices that could not record GPS data to mitigate further confidentiality concerns that may stem from location tracking. Developing a secure, automated data collection pipeline was also integral to a feasible and scalable Fitbit implementation. Without automated data logging or an application that accesses the Fitbit application programming interfaces, data can only be visualized on the Fitbit app or manually downloaded, neither of which represents a feasible approach to large-scale data management.

In summary, findings from this study support the utility of Fitbits as an acceptable and feasible method of objective data collection from youth experiencing acute and chronic pain. Future directions should explore how this wearable technology, coupled with passive sensing and ecological momentary assessment, may be leveraged toward continuous and valid assessment of pain-impacted functional measures and biopsychosocial correlates in pediatric pain populations [42,43]. The sleep, activity, and heart rate can be correlated to medications and clinical pain interventions in the future, as well as perioperative adverse events. Youth self-monitoring of physical activity and sleep patterns may also better promote patient-centered collaborative care between providers and youth with pediatric pain. Our findings also underscore the importance of patient confidentiality and data privacy when implementing wearable devices. We hope our preliminary research methodology of collecting and analyzing data from a wearable device in youth with acute and chronic pain treated in outpatient and inpatient settings can help future pediatric pain studies answer specific questions and test hypotheses.

## Supplementary material

10.2196/59074 Multimedia Appendix 1 Supplementary materials.

## Abbreviations

DOS: date of surgery
HIPAA: Health Insurance Portability and Accountability Act
IIPT: intensive interdisciplinary pain treatment
IRB: Institutional Review Board
REDCap: Research Electronic Data Capture
SOP: standard operating procedure
