# Supplementary material for: Evaluating Fitbits for Assessment of Physical Activity and Sleep in Pediatric Pain: Feasibility and Acceptability Pilot Study
Source: JMIR Form Res. 2025 Jul 30;9:e59074. doi: 10.2196/59074 (PMC12310148; doi:10.2196/59074)
Supplement: Multimedia Appendix 1 [file formative-v9-e59074-s001.pdf]

SUPPLEMENTAL INFORMATION

Table of Contents:

I. Sample Recruitment Flyer for Acute Pain Sample

II. Standard Operating Procedure

III. Figure 1

IV. Figure 2

I. Sample Recruitment Flyer for Acute Pain Sample

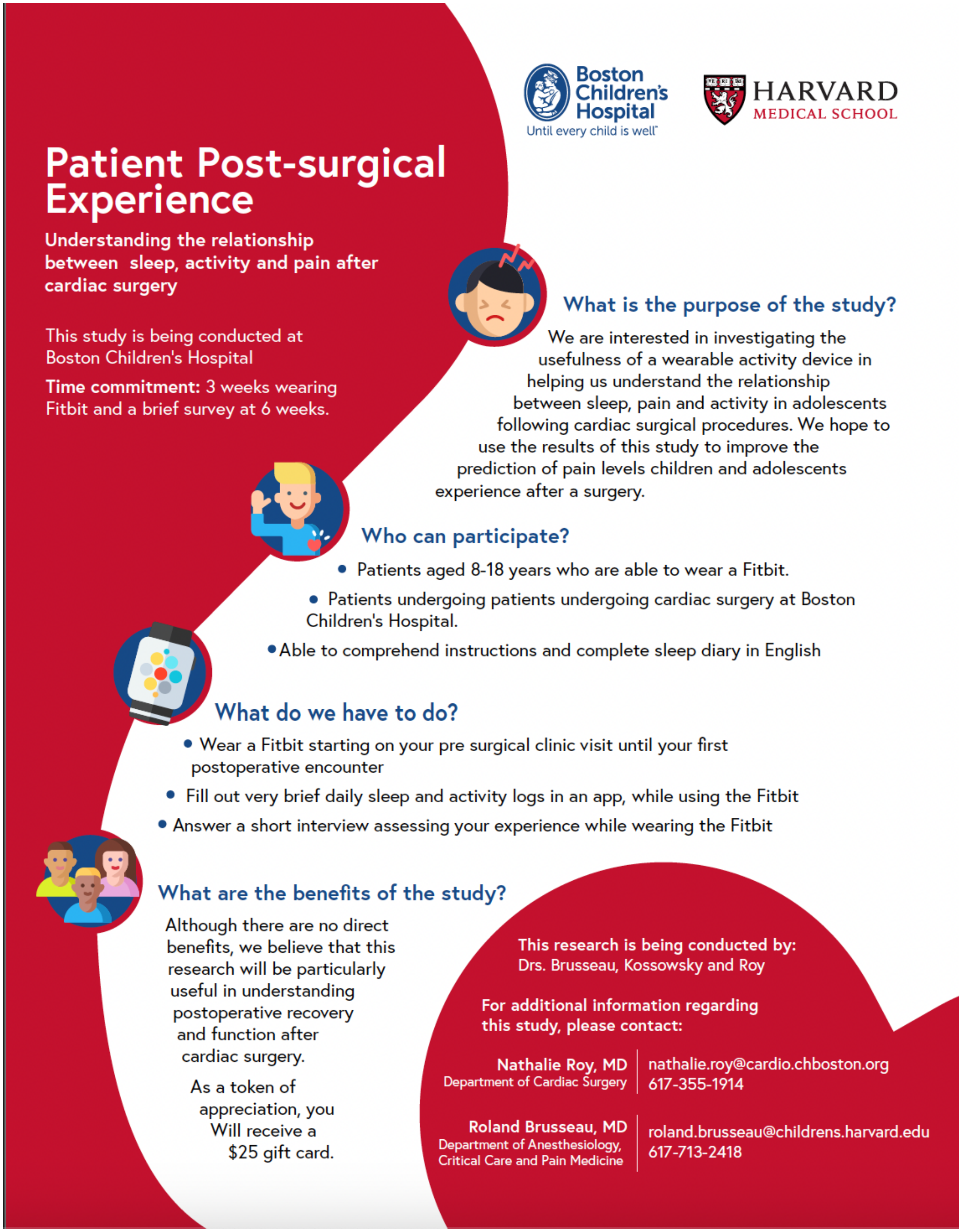

II. Standard Operating Procedure

Charging Your Fitbit:

1. First, open your Fitbit box and make sure you have (1) the Fitbit Inspire 2 Tracker with wrist band, (2) a gray charging cable, and (3) an alternate sized wrist band.

(1)

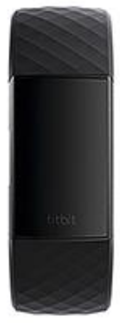

(2)

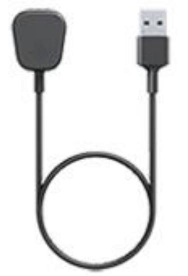

(3)

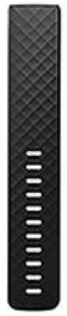

2. Your Fitbit might need to be charged. Please connect the gray charging cable to any standard USB power adapter. Then “clip” the gray charging adapter onto your Fitbit.

Example of a USB power adapter

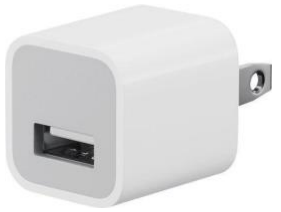

Charging adapter “clipped” onto watch

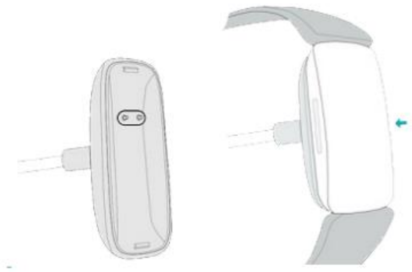

3. Charging fully takes about 1-2 hours. While the tracker charges, you can press the two side buttons to check the battery level. A fully-charged tracker shows a solid battery icon with a smile. A fully charged Fitbit device should last for 7 days before it needs to be charged again.

Fully charged Fitbit icon

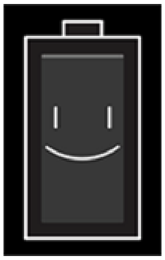

Setting Up Your Fitbit Watch:

1. Download the Fitbit application from the application store on the smartphone device that you will be using throughout the duration of the research study. Open the app.

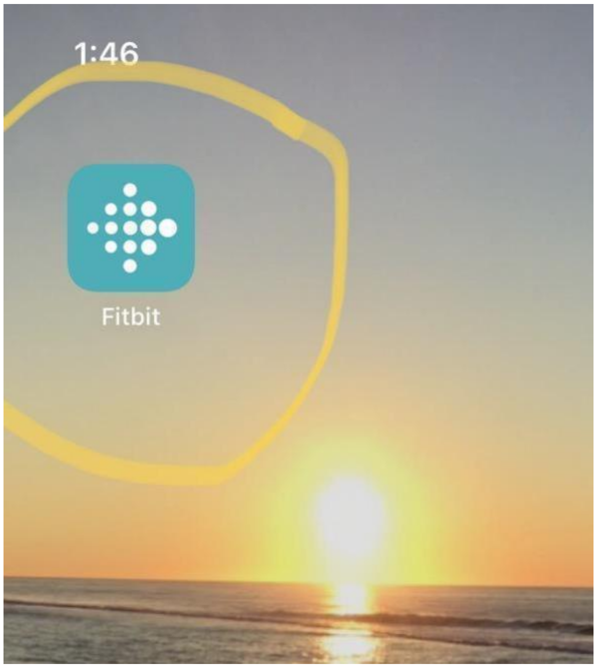

2. Select "OK" for Fitbit to use Bluetooth (this is how the watch will connect with your phone and app). You do NOT need to enable any other features (location, notifications, etc.)

3. S[illegible]

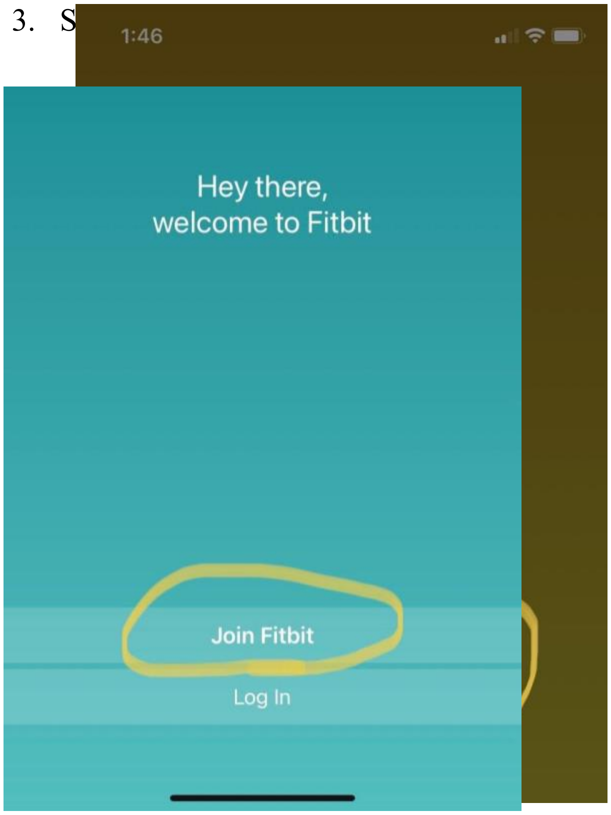

4. Select “Inspire 2” as the device

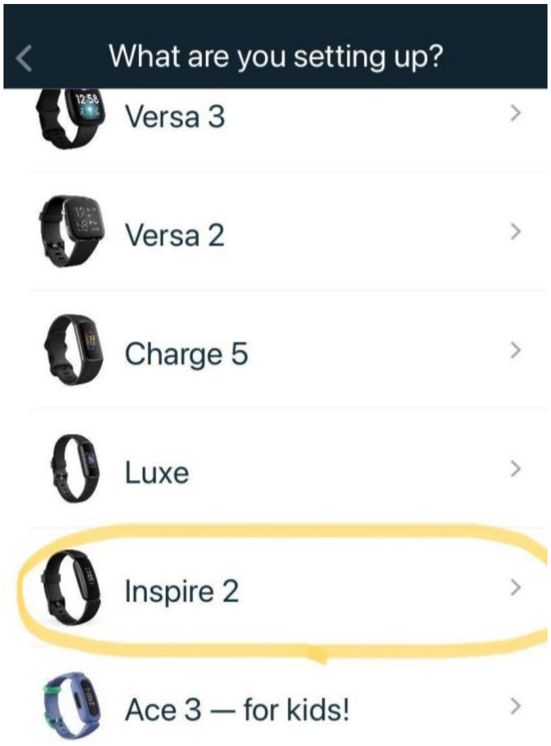

5. Select “Set Up”

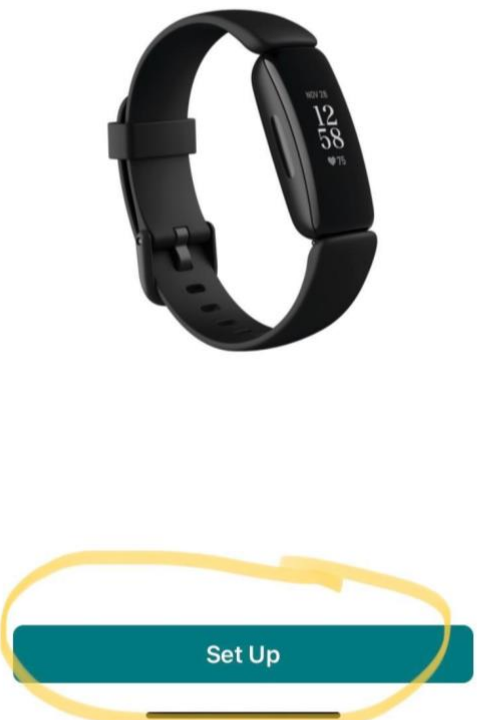

6. Under First Name, enter “Study Participant”

Under Last Name, enter “BCH”

Under Email and Password, enter the Email and Password given to you by the research team. If you do not remember your Email and/or Password, please text/call 617-877-0014 or email dartlab@childrens.harvard.edu

Please DO NOT input any of your actual information here!

Select “I agree to the Fitbit Terms of Service” and then select “Next”

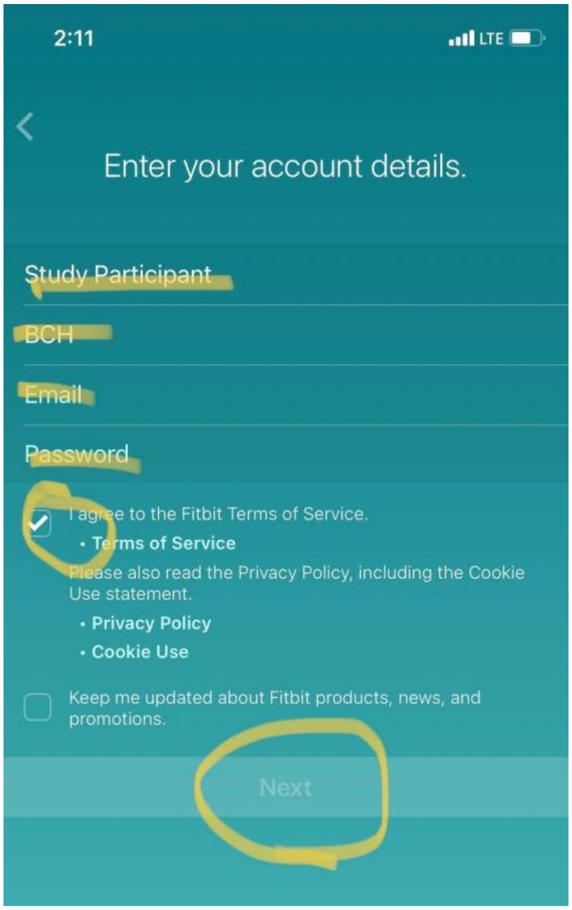

7. Select “I Agree”

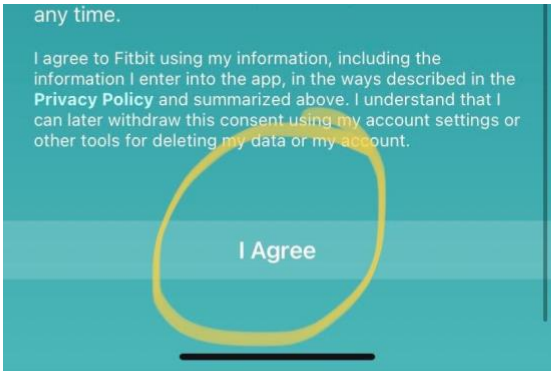

8. Under Birthday, leave answer as is
Under Height, leave answer as is
Under Weight, leave answer as is
Under Sex, select either Male or Female
(Note: it does not matter which you select)

Please DO NOT input any of your actual information here!

Select "Create Account"

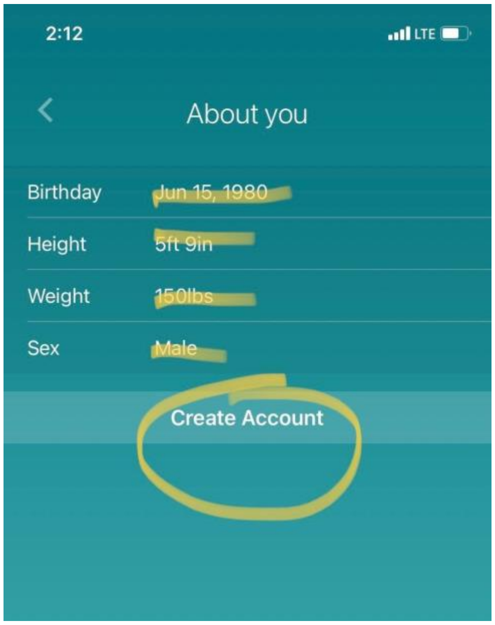

9. Select "I Accept"

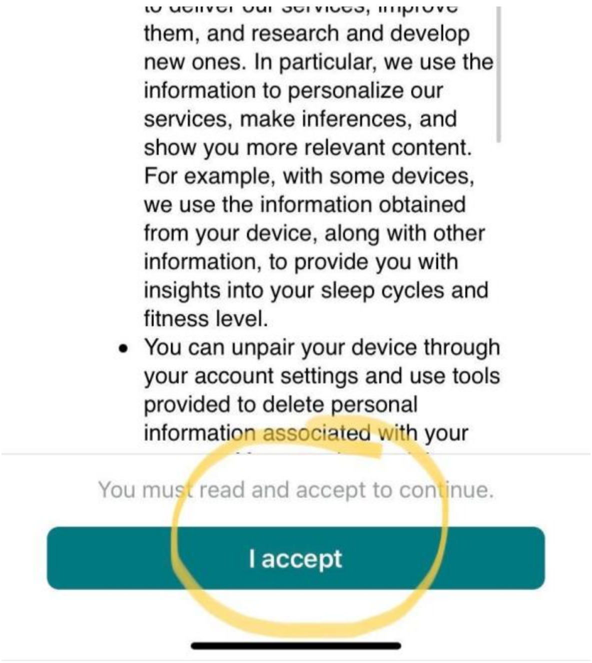

10. Make sure your Fitbit is connected to the charging cable and is plugged into an outlet while connecting

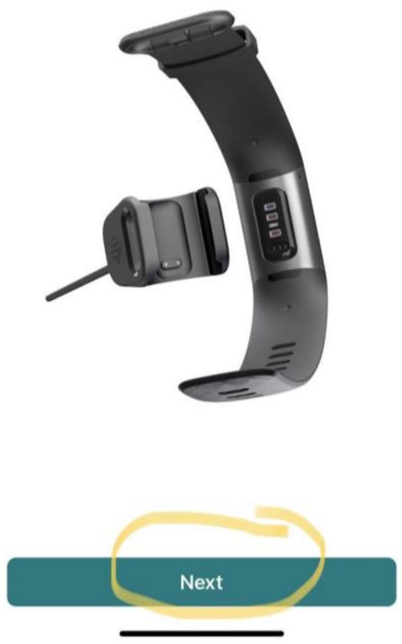

11. When your phone finds your Fitbit watch, it will send a four digit code that will display on your watch. Enter this code in the app.

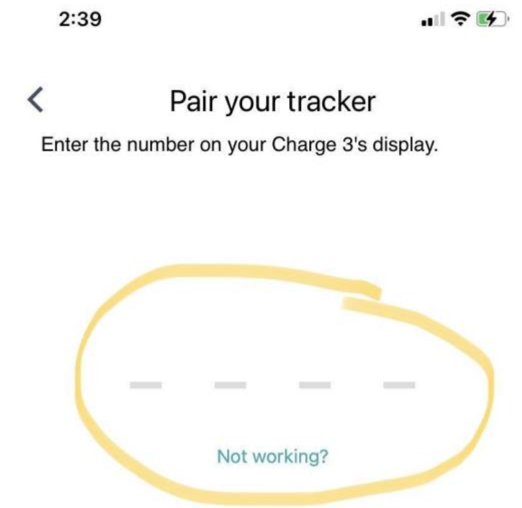

12. Follow through with the remaining instructions

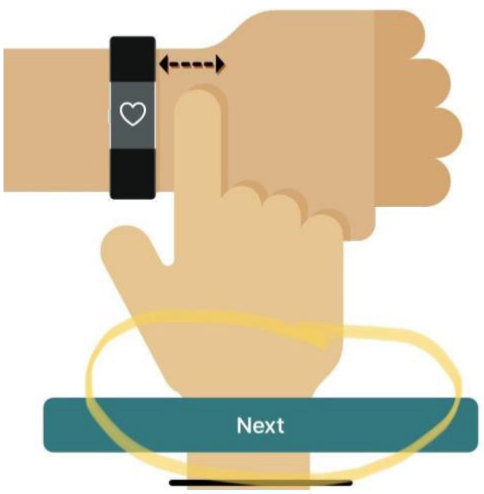

13. Select "Done"

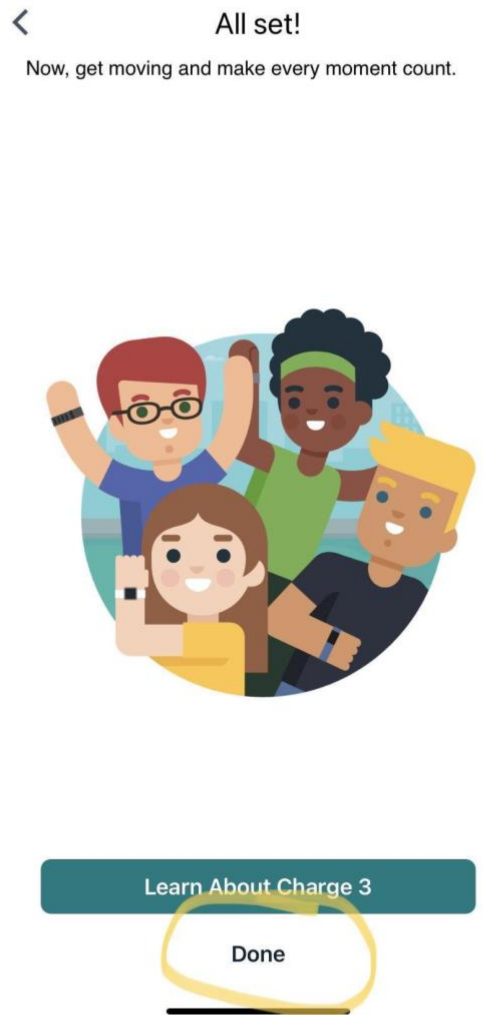

<u>Syncing the Fitbit Charge 3 Watch:</u>

There are two approaches to syncing data from the Fitbit Charge 3 Watch:

1. Data should sync automatically when the Fitbit app is in range of the Fitbit
2. To manually sync data from the Fitbit Charge 3, follow the steps below every 3-5 days to ensure data is properly synced

1) Go into the Fitbit application

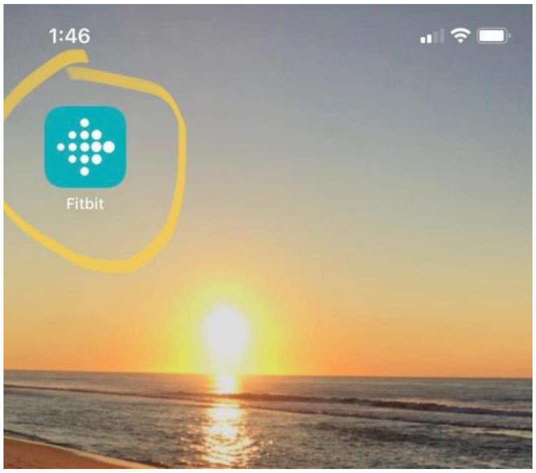

2) Go to your account in the top left corner

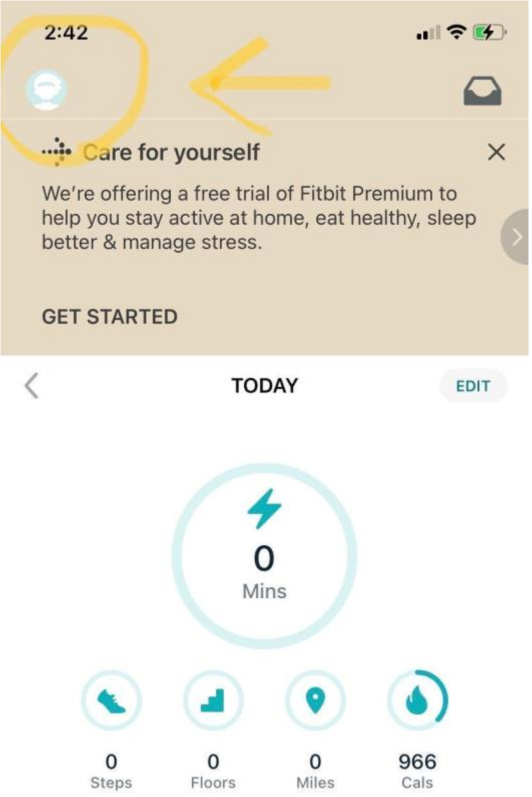

3) Select "Charge 3"

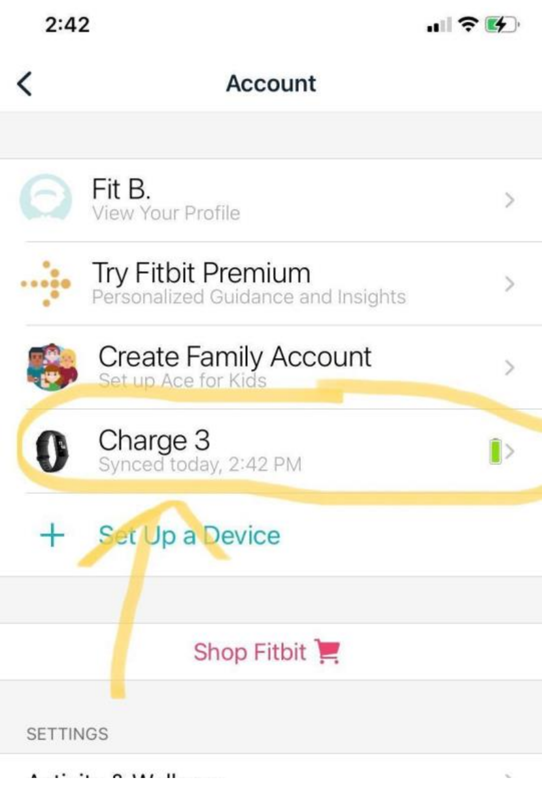

4. Select "Sync Now"

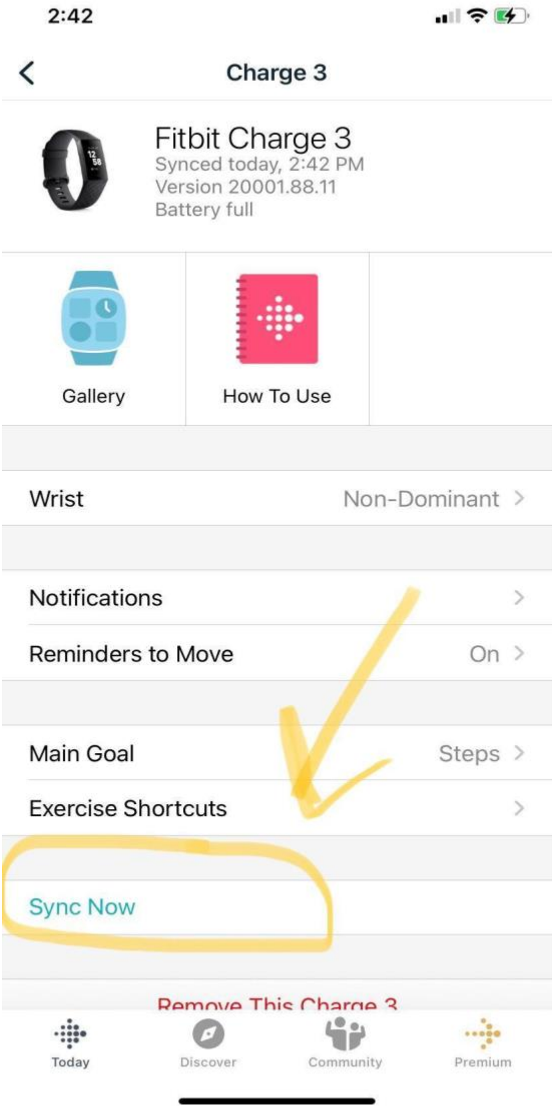

5. You should now see it says "Syncing…"

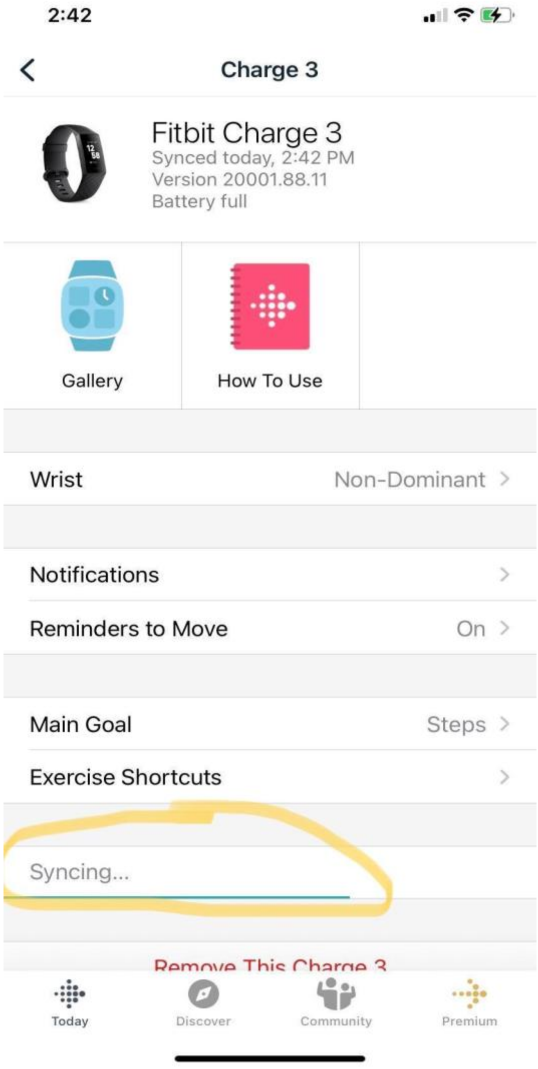

6. You should notice that the top of the page has updated to say "Synced today, 2:42 PM" which will show the time you last synced the device

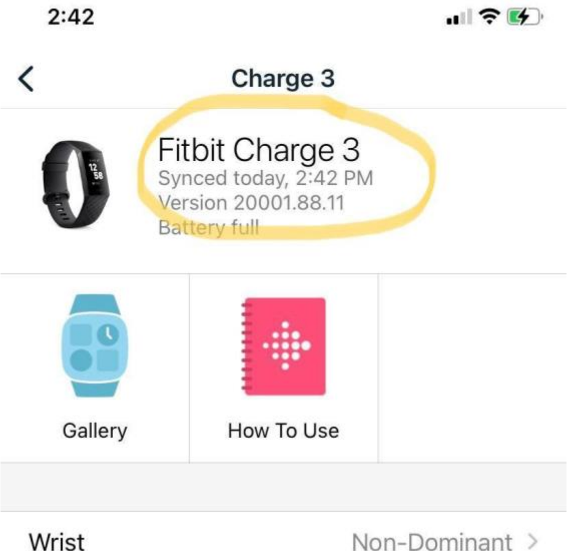

III. Figure 1

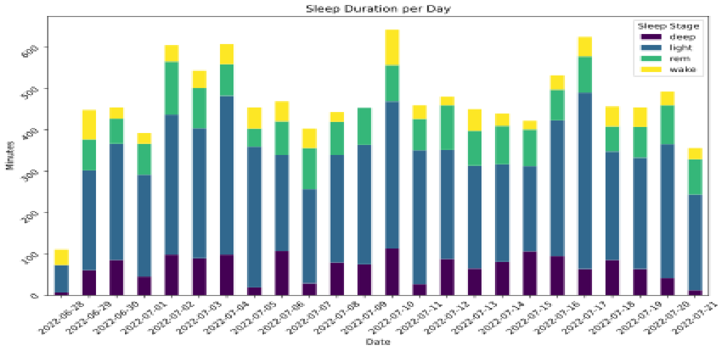

1a. Sleep Duration per Day

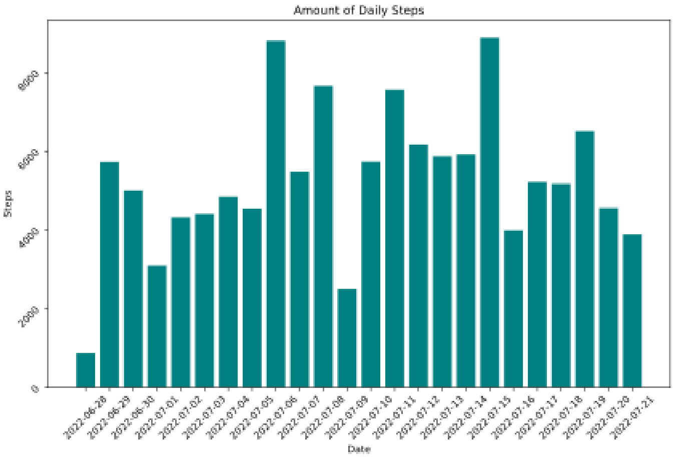

1b. Amount of Daily Steps

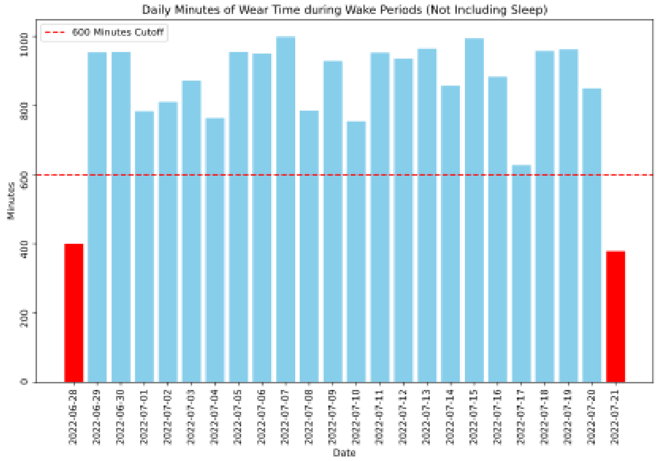

1c. Daily Minutes of Wear Time During Wake Periods (Not Including Sleep)

Figure 1. Sample Data Visualization Report Generated by Automated Fitbit Data Pipeline

Note. The top figure presents a sample participant's sleep duration, including sleep stages. The middle figure presents a sample participant's number of steps per day. The bottom figure provides information about participant compliance, such as the number of minutes per day with Fitbit worn.

IV. Figure 2

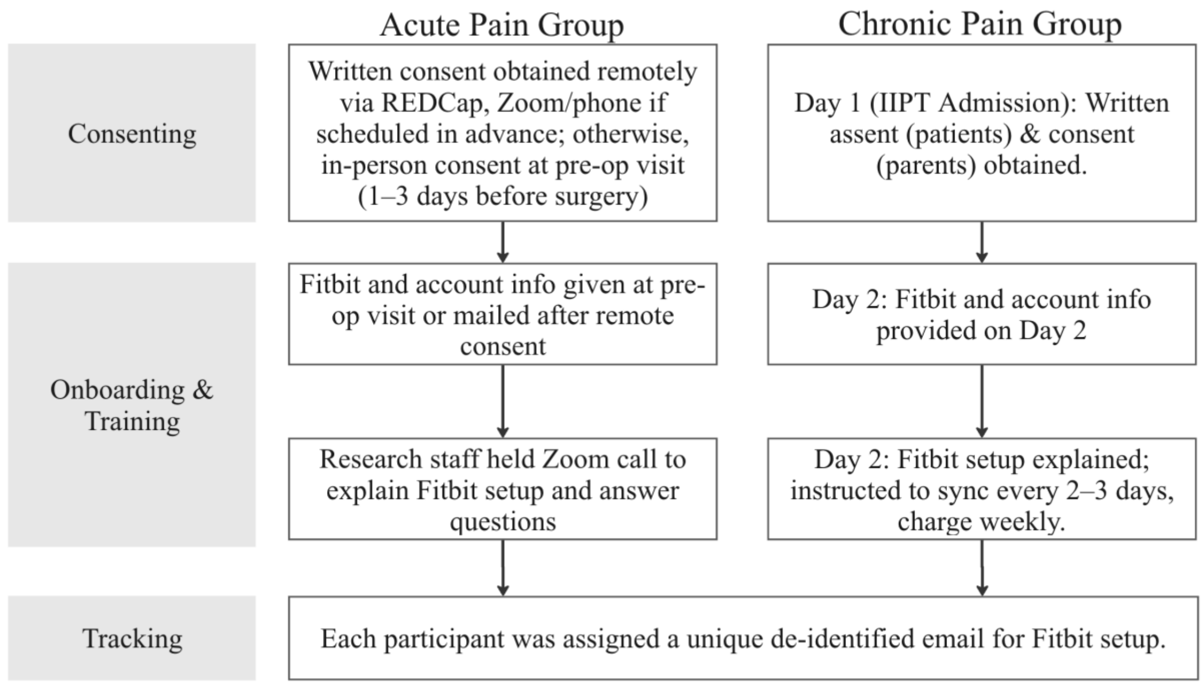
Acute Pain Group
Chronic Pain Group
Consenting
Written consent obtained remotely via REDCap, Zoom/phone if scheduled in advance; otherwise, in-person consent at pre-op visit (1–3 days before surgery)
Day 1 (IIPT Admission): Written assent (patients) & consent (parents) obtained.
Onboarding & Training
Fitbit and account info given at pre-op visit or mailed after remote consent
Day 2: Fitbit and account info provided on Day 2
Research staff held Zoom call to explain Fitbit setup and answer questions
Day 2: Fitbit setup explained; instructed to sync every 2–3 days, charge weekly.
Tracking
Each participant was assigned a unique de-identified email for Fitbit setup.
